# Construction and Validation of a T Cell Exhaustion–Related Prognostic Signature in Cholangiocarcinoma

**DOI:** 10.1155/ijog/8823837

**Published:** 2025-03-10

**Authors:** Changshi Qian, Yuqiao Sun, Yihuai Yue

**Affiliations:** ^1^Department of Hepatopancreatobiliary Surgery, Affiliated Hospital of Yanbian University, Yanji, China; ^2^Department of Surgery, Medical College of Yanbian University, Yanji, China

**Keywords:** cholangiocarcinoma, immune cell infiltration, machine learning, prognosis, T cell exhaustion, weighted coexpression network analysis

## Abstract

**Objective:** T cell exhaustion (TEX) is a critical determinant of immune resistance. This study was performed to investigate the key genes linked to TEX in cholangiocarcinoma (CCA) and construct a TEX-associated gene signature to forecast the prognosis of patients with CCA.

**Methods:** Based on the expression data acquired from the E-MTAB-6389 dataset, the TEX-related modules and module genes were identified using weighted coexpression network analysis (WGCNA). Subsequently, a TEX-related prognostic signature was built by using the univariate and least absolute shrinkage and selection operator (LASSO) Cox regression analysis. The immune cell infiltration in each CCA sample was evaluated using the single-sample gene set enrichment analysis (ssGSEA) package, followed by single-cell RNA sequencing (scRNA-seq) analysis. Furthermore, the expression of TEX-related genes in the gene signature was experimentally validated in CCA cells by quantitative reverse transcriptase polymerase chain reaction (qRT-PCR) and western blot analysis.

**Results:** A total of 15 TEX-associated modules and 23 module genes were identified. Then, a four-gene signature related to TEX was established, containing Palladin, Cytoskeletal Associated Protein (*PALLD*), Member RAS Oncogene Family (*RAB31*), ADAM Metallopeptidase With Thrombospondin Type 1 Motif 2 (*ADAMTS2*), and *WISP1*, which could predict prognosis of patients with CCA. Moreover, neutrophils, endothelial cells, B cells, and T cells exhibited significant infiltration in CCA samples, and these four TEX-related genes were both significantly positively correlated with T cells, endothelial cells, and B cells while negatively correlated with neutrophils. Moreover, a total of 13 cell types were annotated after scRNA-seq analysis. Notably, *RAB31* was mainly highly expressed in monocytes, macrophages, DC2 (Dendritic Cells 2), and DC3 (Dendritic Cells 3), and *PALLD*, *ADAMTS2*, and *WISP1* were mainly overexpressed in fibroblasts. Furthermore, experimental validation revealed that the expression levels of *PALLD*, *RAB31*, *ADAMTS2*, and *WISP1* were consistent with the trend results of bioinformatics analysis.

**Conclusion:** A prognostic signature was developed by four TEX-related genes, including *PALLD*, *RAB31*, *ADAMTS2*, and *WISP1*, which might be a powerful predictor for the prognosis of patients with CCA. These TEX-related genes were related to the infiltration of neutrophils, endothelial cells, B cells, and T cells in CCA.

## 1. Introduction

Cholangiocarcinoma (CCA) is a rare malignancy originating in the bile ducts, comprising approximately 3% of all gastrointestinal malignancies [1, 2]. Surgery remains the primary treatment for patients diagnosed with CCA at an early stage; however, postoperative recurrence rates are high [3]. Immunotherapy with immune checkpoint inhibitors (ICIs) is being investigated to improve CCA therapy [4, 5]. The programmed death 1 (PD1)/programmed death ligand-1 (PD-L1) pathway is abnormally activated in some CCA patients, which prompted calls for targeted therapy at immune checkpoint blockade (ICB) [6, 7]. Multiple variables, containing the tumor microenvironment (TME), can impact ICI efficacy, and numerous biomarkers can accurately predict patient prognosis [8]. However, we know very little about the TME of CCA, and improved prognostic indicators are immediately required.

Accumulating evidence has revealed that TME is important for cancer progression and treatment response [9, 10]. Tumor-infiltrating T cells represent a crucial element of the TME, serving a fundamental role in the recognition and eradication of tumor cells [11]. Tumor-infiltrating T cells are also an essential component of the adaptive immune system and essential for the efficacy of current cancer immunotherapies, encompassing ICIs and adoptive cell therapies [12]. Additionally, tumor-infiltrating T cells are a hopeful sign for forecasting the prognosis and immunotherapy for CCA patients [13]. However, cancer progression frequently leads to T cell exhaustion (TEX), a state of diminished function marked by the gradual loss of T cell effector abilities and self-renewal potential [14]. It is reported that TEX poses a formidable obstacle to current anticancer immunotherapy, as they lose the ability to produce antitumor cytokines, along with compromised proliferation and cytotoxicity [15]. Previous studies suggest that T cells in the intermediate stages of exhaustion show the most satisfactory response to treatment with ICIs, which can effectively restore T cell cytotoxic functions [16, 17]. Therefore, we have focused more on TEX; reversing TEX may be the key to improving the objective response rate of CCA receiving immunotherapy.

Weighted coexpression network analysis (WGCNA) is widely applied for the discovery of disease biomarkers [18]. Machine learning is a special class of algorithms that enable computers to learn from data and make predictions autonomously, which contributes to the investigation of disease mechanisms and clinical diagnosis [19, 20]. Xu et al. established a prognostic model for breast cancer based on genes associated with disulfidptosis, cuproptosis, and ferroptosis through machine learning– and WGCNA-mediated double analysis [21]. Lin et al. also constructed an endoplasmic reticulum stress–related prognostic model for endometrial cancer based on WGCNA and machine learning algorithms [22]. Therefore, this study attempted to explore the key genes linked to TEX in CCA and to construct a TEX-associated gene signature to predict the prognosis of CCA patients through WGCNA combined with machine learning. Moreover, we analyzed the immune cell infiltration of CCA samples and its association with TEX-related genes in the gene signature. Furthermore, the expression of signature genes was experimentally validated in CCA cells. Our findings will furnish a theoretical foundation for the clinical decision-making of CCA.

## 2. Materials and Methods

### 2.1. Data Source

The E-MTAB-6389 dataset was acquired from the European Molecular Biology Laboratory's European Bioinformatics Institute (EMBL-EBI) database (https://www.ebi.ac.uk/). This dataset contained 78 tumor tissue samples and was used as the training dataset. The gene expression dataset GSE107943 was obtained from the National Center for Biotechnology Information (NCBI) Gene Expression Omnibus (GEO) database, which was generated on the GPL18573 platform and used for external verification of the prognostic signature. In addition, the single-cell RNA sequencing (scRNA-seq) dataset GSE138709 was acquired from the GEO database, which contained five tumors and three normal samples.

### 2.2. Identification of TEX-Related Modules and Module Genes

Using the TEX-related pathway gene set [23] from the Molecular Signatures Database (MSigDB) [24], combined with the expression data from the E-MTAB-6389 dataset, the pathway activity scores were determined using the gene set variation analysis (GSVA) package [25] (Version 1.50.1) in R (Version 4.3.2). Subsequently, the WGCNA package [26] (Version 1.72-5) in R (Version 4.3.2) was employed to conduct a scale-free WGCNA. The key modules were identified by comparing the correlation between modules and sample groups. In this study, the soft threshold was determined as *β* = 4 according to the principle of a scale-free network, which was selected based on scale independence and mean connectivity. The modules were then identified with the parameters of minModuleSize = 25, mergeCutHeight = 0.4, and Seed = 20. The key modules with higher correlation with TEX-related pathway activity scores were chosen with the cutoff value of *p* < 0.05. By further comparing the association between genes and modules as well as the correlation between genes and TEX-related pathway scores, TEX-associated module genes were obtained.

### 2.3. Construction of a Prognostic Model

Based on the clinical survival information of patients and the expression data of TEX-associated module genes, univariate Cox regression analysis was conducted to select prognosis-related genes using the survival package [27] (Version 3.5-8) in R (Version 4.3.2) with the threshold value of *p* < 0.05. According to the patient survival data, a total of 75 samples were finally screened with survival time greater than 0. Then, the samples were randomly divided into a training set (35 samples) and a testing set (40 samples). Utilizing the training set, the least absolute shrinkage and selection operator (LASSO) Cox regression analysis was conducted using the glmnet package (Version 4.1-8) with a random seed of 2022. The 10-fold cross-validation was carried out using the cv.glmnet function. Then, a prognostic model was constructed by calculating the following formula: riskscore = ∑_*n*=1_^*n*^coefi∗*Xi*, where *i* represents the regression coefficient and *X* indicates the expression value of the gene. After determining the risk score of each sample, they were categorized into high-risk (HR) and low-risk (LR) groups according to the median value. The prognostic value of this model was evaluated using the Kaplan–Meier survival curve and receiver operating characteristic (ROC) curve analyses. Furthermore, the predictive value of the model was internally verified based on the testing set and externally validated with the GSE107943 dataset.

### 2.4. Immune Infiltration Analysis

The infiltration levels of 23 kinds of immune cells in each CCA sample were analyzed using the single-sample gene set enrichment analysis (ssGSEA) function [28] in R (Version 4.3.2), followed by assessing their differences between HR and LR groups using a *t*-test. Differential immune cells between the two groups were screened with the cutoff value of *p* < 0.05. The interplay between differential immune cells and the genes in the prognostic model was explored by Pearson's correlation analysis.

### 2.5. scRNA-seq Analysis

Based on the GSE138709 dataset, the Seurat package [29] (Version 4.4.0) was employed to conduct dimensionality reduction and clustering. Assisted annotation of cells obtained from cluster analysis was performed by using CellMarker (Version 2.0). Then, the four model genes were mapped in the GSE138709 dataset.

### 2.6. Cell Culture

Two CCA cell lines, HuH28 (Xiamen Yimo Biotechnology Co. Ltd., Xiamen, China) and HuCCT1 (Wuhan Punuosi Life Science and Technology Co. Ltd., Wuhan, China), as well as a normal intrahepatic biliary epithelial cell line, HIBEC (Wuhan Punuosi Life Science and Technology Co. Ltd., China), were cultivated in the RPMI-1640 medium (No: A5669701, Gibco, Grand Island, NY, United States) with the addition of 10% fetal bovine serum (FBS; No: A5669701, Gibco) and 1% penicillin/streptomycin (No: SV30010, Hyclone, Logan, UT, United States). These cells were then maintained in a 37°C, 5% CO_2_ incubator. Routine passage was performed when reaching approximately 90% confluence.

### 2.7. Quantitative Reverse Transcriptase Polymerase Chain Reaction (qRT-PCR)

The extraction of total RNA from cells was carried out using TRIzol reagent (No: 15596018, Invitrogen, Carlsbad, CA, USA), followed by the synthesis of cDNA through the FastKing RT Kit (No: KR118-02, Tiangen, Beijing, China). The target gene expression was then detected via qRT-PCR using SYBR Green polymerase chain reaction (PCR) Master Mix (No: A4004M, Xiamen Life Interconnect Technology Co. Ltd., Xiamen, China). The primers for gene amplification are displayed in Table 1. The cycling conditions were as follows: 95°C for 3 min, 40 cycles of 12 s at 95°C, and 40 s at 62°C. Glyceraldehyde-3-phosphate dehydrogenase (GAPDH) served as an internal control. Relative quantification of gene expression was determined using the 2^–ΔΔCt^ method [30].

### 2.8. Western Blot Analysis

Total protein was extracted from cells using radioimmunoprecipitation (RIPA) lysis buffer. After quantification by BCA (bicinchoninic acid) Kit (No: PC0020, Solarbio, Beijing, China), the isolated proteins (25 *μ*g of each sample) were separated by sodium dodecyl sulfate polyacrylamide gel electrophoresis (SDS-PAGE), and then target proteins were transferred onto polyvinylidene fluoride (PVDF) membrane (No: FFP24, Beyotime, Shanghai, China). After blocking with 5% skimmed milk, the membranes were marked with the primary antibodies, including anti-ADAMTS2 (ADAM Metallopeptidase With Thrombospondin Type 1 Motif 2) antibody (1:1000; No: DF9175, Affinity, United States), anti-PALLD (Palladin, Cytoskeletal Associated Protein) antibody (1:1000; No: DF9731, Affinity, United States), anti-RAB31 (Member RAS Oncogene Family) antibody (1:1000; No: DF4401, Affinity, United States), anti-WISP1 antibody (1:1000; No: DF12503, Affinity, United States), and anti-GAPDH (1:2500; No: ab181602, Abcam, United States) at 4°C overnight. Then, the membranes were incubated with the horseradish peroxidase (HRP)-conjugated goat anti-rabbit IgG antibody (1:2000). Proteins on the PVDF membrane were visualized using an ECL (enhanced chemiluminescence) solution (No: P1000, Applygen, Beijing, China). GAPDH was used as the loading control.

### 2.9. Statistical Analysis

All data were reported as mean ± standard deviation (SD), and one-way analysis of variance (ANOVA) was employed to evaluate the differences among groups. GraphPad 8.0 software (GraphPad Software, San Diego, CA) was utilized for all statistical analyses, where *p* < 0.05 was the threshold for significance.

## 3. Results

### 3.1. Identification of TEX-Associated Module Genes

**Figure 1:**
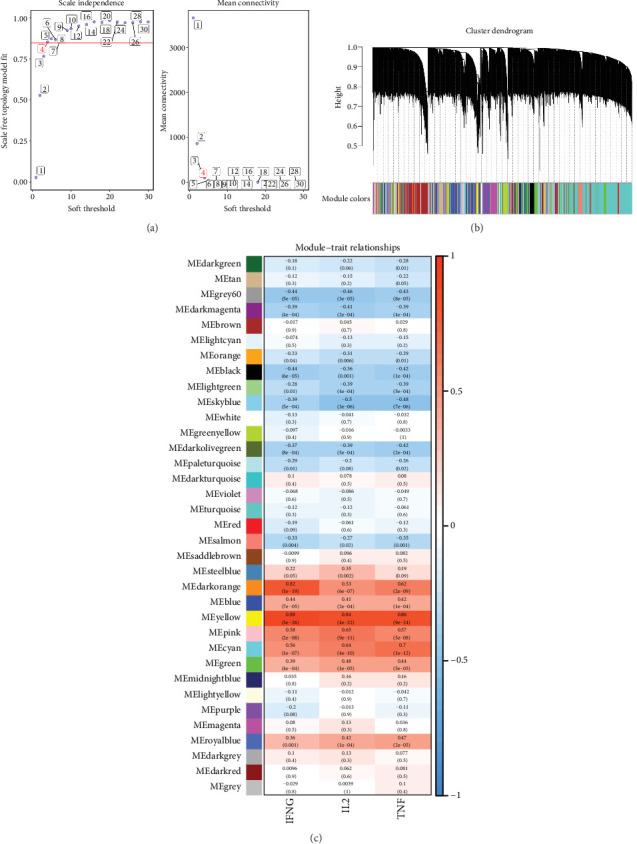
WGCNA for the identification of TEX-associated modules. (a) Soft threshold selection based on scale independence and mean connectivity. (b) Coexpression clustering of gene modules. Different modules are given different colors, and gray indicates that genes are outside all modules. (c) The module–trait relationships. Blue is negatively correlated, and red is positively correlated. WGCNA, weighted gene coexpression network analysis; TEX, T cell exhaustion.

By comparing the correlation between modules and TEX-related pathways, key modules were identified. When the soft threshold was set to 4, the constructed network conformed to a scale-free distribution (Figure 1(a)). With mergeCutHeight = 0.4, 35 coexpression modules were screened (Figure 1(b)). Subsequently, the correlation between each module and TEX-related features was evaluated, and the heat map of module–trait relationships is shown in Figure 1(c). Based on *p* < 0.05, 15 key modules such as green-yellow and turquoise were identified. The association between genes and modules, as well as between genes and TEX pathway scores, was then analyzed, resulting in the selection of 23 TEX-associated module genes, containing *ADAMTS2*, *BASP1*, *CHSY1*, *COTL1*, *FCGR1A*, *FCGR1B*, *FSTL1*, *GPNMB*, *HAS2*, *HLA-C*, *LAPTM5*, *LXN*, *MAX*, *PALLD*, *PCED1B*, *RAB31*, *RGS10*, *SAMD9L*, *TIMP1*, *TMEM200A*, *TMSB4XP4*, *TRPV2*, and *WISP1*.

### 3.2. Construction and Validation of Prognostic Signature

**Figure 2:**
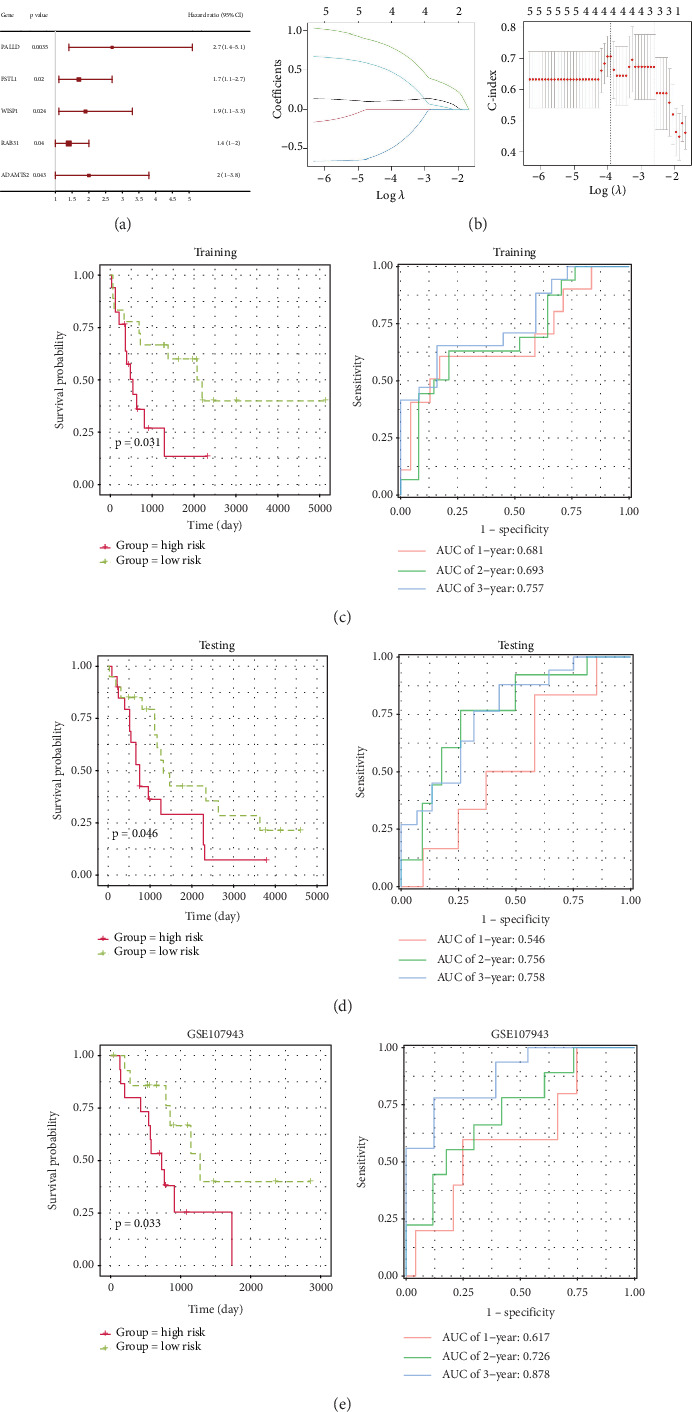
Construction and validation of a TEX-related prognostic model. (a) Univariate Cox regression analysis for the identification of TEX-associated prognostic genes. (b) The coefficient spectrum and optimized lambda determined in the LASSO regression model. (c) Survival and ROC curves showed the model performance based on the training set. (d) Survival and ROC curves showed the model performance based on the testing set. (e) Survival and ROC curves showed the model performance based on the external validation dataset GSE107943. LASSO, least absolute shrinkage and selection operator; ROC, receiver operating characteristic; AUC, area under the ROC curve.

By combining the clinical survival information of patients, univariate Cox regression analysis revealed that five of the 23 TEX-associated module genes were significantly related to the prognosis of patients (*p* < 0.05, Figure 2(a)). In the training dataset, LASSO Cox regression analysis identified four TEX-related prognostic genes for establishing the prognostic model (Figure 2(b)). Four genes were *PALLD*, *RAB31*, *ADAMTS2*, and Cellular Communication Network Factor 4 (*WISP1*, also known as *CCN4*). This model was constructed with the following formula: risk score = *ADAMTS*2∗0.11096 + *PALLD*∗0.77504 − *RAB*31∗0.47375 + *WISP*1∗0.45876. Moreover, the Kaplan–Meier survival curve analysis showed that the HR group had a substantially lower survival rate than in the LR group in the training set (*p* = 0.031), and the ROC curve showed that the AUC (area under the curve) value of the model in predicting 1-, 2-, and 3-year survival rates was all greater than 0.68 (Figure 2(c)). In the testing set, the survival rate of the HR group was significantly lower than that of the LR group (*p* = 0.046), and the 3-year AUC value was 0.758 (Figure 2(d)). In the external validation dataset GSE107943, the HR group exhibited a decreased survival rate in comparison to the LR group (*p* = 0.033), and the 1-, 2-, and 3-year AUC values were all greater than 0.617 (Figure 2(e)). These data indicated that the signature had a high predictive power for the prognosis of CCA patients.

### 3.3. Immune Infiltration Analysis

**Figure 3:**
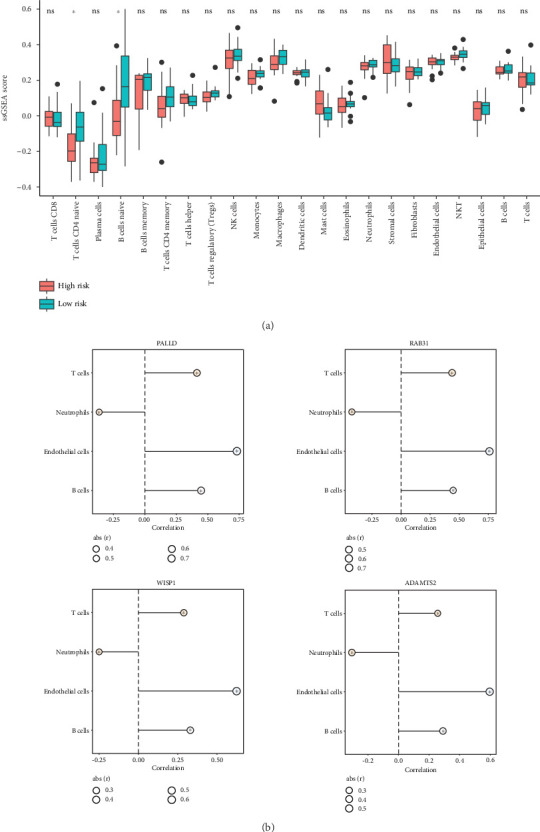
The TEX-related prognostic model was related to immune cell infiltration. (a) Comparison of infiltration proportion of multiple immune cells between high- and low-risk groups. (b) The correlation between differential immune cells and four model genes (*ADAMTS2*, *PALLD*, *RAB31*, and *WISP1*).

Using the ssGSEA method, the infiltration of 16 immune cells was observed in CCA samples. Analysis of differences using a *t*-test indicated that the HR group had notably lower neutrophil infiltration compared to the LR group but significantly higher infiltration of endothelial cells, B cells, and T cells (*p* < 0.05, Figure 3(a)). Moreover, the correlation analysis demonstrated that the four TEX-related genes (*ADAMTS2*, *PALLD*, *RAB31*, and *WISP1*) in the signature were significantly associated with four types of differential immune cells, with a negative correlation observed between the four genes and neutrophils and a positive correlation with the other three immune cell types (Figure 3(b)).

### 3.4. Correlation Between the Expression of Neutrophil Extracellular Trap (NET) Gene and Four Model Genes and scRNA-seq Analysis

**Figure 4:**
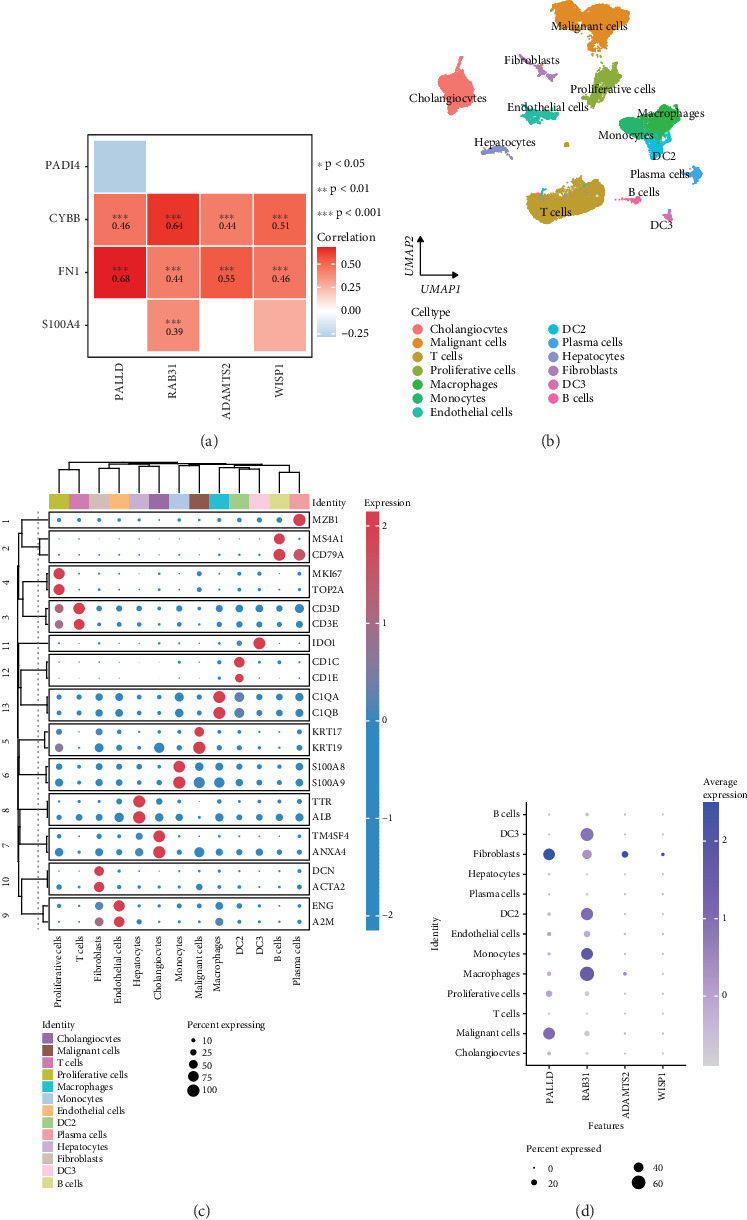
Correlation between the expression of the NET gene and four model genes and scRNA-seq analysis. (a) Correlation between the expression of the NET gene (*ELANE*, *MPO*, *S100A4*, *FN1*, *CYBB*, and *PADI4*) and four model genes. ⁣^∗∗∗^*p* < 0.001. (b) Cluster annotation. (c) Dotplot of expression of marker genes in 13 cell types. (d) Dotplot of expression of four model genes in 13 cell types.

Besides, the correlation between the expression of the NET gene (*ELANE*, *MPO*, *S100A4*, *FN1*, *CYBB*, and *PADI4*) and the four TEX-related genes in the signature was explored based on the E-MTAB-6389 dataset. As shown in Figure 4(a), these four TEX-related genes were both significantly positively correlated with *FN1* and *CYBB*. Moreover, a total of 13 cell types were annotated, containing cholangiocytes, malignant cells, T cells, proliferative cells, macrophages, monocytes, endothelial cells, DC2 (Dendritic Cells 2), plasma cells, hepatocytes, fibroblasts, DC3 (Dendritic Cells 3), and B cells (Figure 4(b)), and the expression level of the marker genes in these cell types is shown in Figure 4(c). Notably, *RAB31* was mainly highly expressed in monocytes, macrophages, DC2, and DC3, and *PALLD*, *ADAMTS2*, and *WISP1* were mainly overexpressed in fibroblasts (Figure 4(d)).

### 3.5. Validation of Signature Gene Expression in CCA Cells

**Figure 5:**
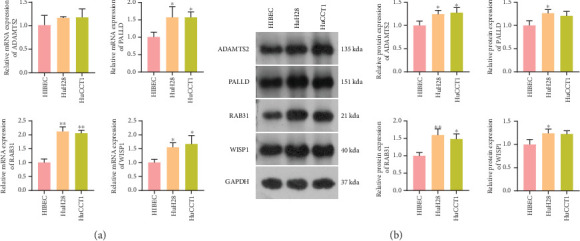
The expression of four model genes (*PALLD*, *RAB31*, *WISP1*, and *ADAMTS2*) in cholangiocarcinoma cells. (a) qRT-PCR was used to detect their expression level. (b) Protein levels of PALLD, RAB31, ADAMTS2, and WISP1 detected by western blot. ⁣^∗^*p* < 0.05 and ⁣^∗∗^*p* < 0.01 compared to HIBEC cells.

To confirm the key role of four signature genes in CCA, we further validated their expression in CCA cells. As shown in Figure 5(a), the *PALLD*, *RAB31*, and *WISP1* mRNA expression levels were significantly elevated in HuH28 and HuCCT1 cells compared to HIBEC cells (*p* < 0.05). Moreover, western blot analysis revealed that both protein levels of PALLD, RAB31, ADAMTS2, and WISP1 in HuH28 and HuCCT1 cells were significantly higher than those in HIBEC cells (both *p* < 0.05, Figure 5(b)). Although the *ADAMTS2* mRNA expression did not show a significant difference, the trend was also upward, which was consistent with the trend of protein expression level. These data suggested that *PALLD*, *RAB31*, *ADAMTS2*, and *WISP1* might be key regulators in the development of CCA.

## 4. Discussion

CCA is a malignant and aggressive disease with a poor prognosis, and the median survival is less than 24 months [31]. TME exerts an impact on CCA progression and metastasis, and the TEX within TME is acknowledged as a critical determinant of immune resistance [32]. In this study, a four-gene signature related to TEX was established, namely, *PALLD*, *RAB31*, *ADAMTS2*, and *WISP1*, which could predict the prognosis of patients with CCA.

WGCNA has become a widely adopted and reliable approach to identify key modules and genes associated with various disease phenotypes [33]. In this study, a total of 23 TEX-associated module genes were identified using WGCNA. Among them, four TEX-related prognostic genes were recognized for establishing a prognostic signature, namely, *PALLD*, *RAB31*, *ADAMTS2*, and *WISP1*. *PALLD* encodes an actin-associated protein crucial for establishing cellular morphology and preserving cytoskeletal organization [34]. *PALLD* expression is intricately linked to the malignant cell motility characteristics exhibited by aggressive cancer cells [35]. *PALLD* is also found to be related to venous invasion and immune capacity in colon cancer [36]. *RAB31*, a RAB5 subfamily member, has been shown to be highly expressed in several cancers and is related to poor prognosis [37]. *ADAMTS2* is a procollagen N-proteinase and functions as a key regulator in cancer progression and prognosis [38]. *WISP1* is part of the CCN protein family that is implicated in various cellular processes and tumor development [39]. High *WISP1* expression is related to CD8+ (cluster of differentiation 8 positive) cell density and primary resistance to ICB in prostate adenocarcinoma [40]. In this study, *PALLD*, *RAB31*, *ADAMTS2*, and *WISP1* may be involved in TEX. Although their specific mechanisms have not been elucidated, a prognostic signature developed by these genes had a high predictive value for the prognosis of patients with CCA. Moreover, qRT-PCR and western blot confirmed the elevated expression levels of *PALLD*, *RAB31*, *ADAMTS2*, and *WISP1* mRNA in CCA cells. Although the *ADAMTS2* mRNA expression did not show a significant difference, the trend was also upward, which was consistent with the trend of protein expression level. The reason for this result may be that qRT-PCR and Western blot are two different experimental techniques that differ in sample processing, detection sensitivity, and specificity, which may lead to differences in detection results. Based on these results, which can provide diagnosis and prognosis prediction for future biomarkers in CCA patients. Moreover, the Kaplan–Meier survival curve analysis showed that the HR group had a substantially lower survival rate than the LR group. Traditional prognostic signatures for CCA patients have offered valuable insights, such as perineural invasion [41], body composition [42], and peritumoral ductular reaction [43]. However, the absence of molecular characteristics limits the comprehensiveness and accuracy of prognostic signatures. As our study has shown, incorporating TEX-related genes can substantially improve the accuracy of overall survival prediction for CCA patients. Thus, this model may contribute to future research on the risk stratification of patients with CCA and the formulation of novel treatment strategies.

A thorough evaluation of immune cell infiltration in the TME can reveal the complex mechanisms that allow cancer to evade immune responses [44]. The immune cell atlas of CCA unveils unique TME and their correlation with prognostic outcomes [45]. To better understand the TME of CCA, we evaluated immune cell infiltration of CCA samples and then assessed its correlation with the prognostic model. We found the infiltration of neutrophils, endothelial cells, B cells, and T cells in CCA samples. Neutrophils are major inflammatory cells in the TME, and their distribution reflects the inflammatory state of CCA [46]. A previous study has shown that immunosuppressive neutrophils expressing *CD10* and *ALPL* contribute to the resistance of anti-PD-1 therapy in hepatocellular carcinoma by facilitating the development of irreversible TEX [47]. Moreover, there is increasing evidence that in CCA, tumor epithelial cells establish complex and interconnected relationships with various stromal components, which remarkably influence the invasive behavior of the tumor [48]. Furthermore, tumor-infiltrating lymphocytes (TILs) exert antitumor activity as the primary immune cells [49]. CD8+ and CD4+ (cluster of differentiation 4 positive) T lymphocytes are the majority of TILs in CCA, which play a crucial role in surveillance and immune responses against tumors and are related to the prognosis of CCA patients [13]. The exhaustion of tumor-specific T cells can hinder the elimination of tumors and ultimately result in immune escape [50]. B cells are common TILs that are essential for the adaptive immunity of cancer. B cells can release immunomodulatory cytokines to influence T cell functions [51]. Increased levels of B cell infiltration are linked to improved overall survival of patients with CCA [15]. This study demonstrated significant correlations between these immune cells and TEX-related genes in the prognostic signature, suggesting that these genes may regulate TEX and CCA prognosis via modulating the infiltration of these immune cell types. These data may provide personalized immunotherapy strategies for CCA patients.

The strength of this study is the first exploration of TEX-related genes in CCA and the construction of the TEX-related gene signature to forecast the prognosis of CCA patients. Despite these, this study has some limitations. First, the key TEX-related genes are identified on the basis of publicly available data, and their roles in CCA are not investigated. Secondly, the mechanisms of TEX-related genes in regulating TEX in CCA are not elucidated. Lastly, the clinical applicability of the prognostic model is not validated in clinical cohorts. More experiments are required to verify our findings.

In conclusion, this study established a stable and accurate prognostic signature based on four TEX-related genes (*PALLD*, *RAB31*, *ADAMTS2*, and *WISP1*), which may be a powerful predictor for the prognosis of CCA patients. These four TEX-related genes are related to the infiltration of neutrophils, endothelial cells, B cells, and T cells in CCA samples. These findings may facilitate the development of personalized immunotherapy strategies for CCA.

## Figures and Tables

**Table 1:** The primer sequences for gene amplification.

**Primers**	**Sequences (5**⁣′**-3**⁣′**)**	**Product length (bp)**
Human *GAPDH* forward	GAAGGTGAAGGTCGGAGTCA	199
Human *GAPDH* reverse	GACAAGCTTCCCGTTCTCAG	
Human *ADAMTS2* forward	GTCCAGAGCAGGGGTACGAG	403
Human *ADAMTS2* reverse	GCGATACACCACATGCACAC	
Human *PALLD* forward	CAACCGAGCAGGACAGAACT	424
Human *PALLD* reverse	CGAAAGTGCTGCATAGCGAC	
Human *RAB31* forward	TGGGGTTGGGAAATCAAGCA	385
Human *RAB31* reverse	GGCACCTATGGATTCAGCGT	
Human *WISP1* forward	GGTGCCCAAACCCAAAACTG	234
Human *WISP1* reverse	ACTGGTCGTTGTCGTTGGAA	

## Data Availability

The raw data of bioinformatics analyses from the current study are openly available in public databases listed within the article. The raw data from experiments are available upon reasonable request.
